# A *De Novo* Whole GCK Gene Deletion Not Detected by Gene Sequencing, in a Boy with Phenotypic GCK Insufficiency

**DOI:** 10.1155/2011/768610

**Published:** 2011-10-20

**Authors:** N. H. Birkebæk, J. S. Sørensen, J. Vikre-Jørgensen, P. K. A. Jensen, O. Pedersen, T. Hansen

**Affiliations:** ^1^Department of Pediatric, Aarhus University Hospital, Skejby, 8200 Aarhus C, Denmark; ^2^Department of Clinical Genetic, Aarhus University Hospital, Nørrebrogade, 8200 Aarhus C, Denmark; ^3^Hagedorn Research Institute, 2820 Gentofte, Denmark; ^4^Novo Nordisk Foundation Center for Basic Metabolic Research, Faculty of Health Sciences, University of Copenhagen, 1350 Copenhagen K, Denmark; ^5^University of Aarhus, 8200 Aarhus C, Denmark; ^6^Steno Diabetes Center, 2820 Gentofte, Denmark; ^7^Faculty of Health Sciences, University of Southern Denmark, 5000 Odense C, Denmark

## Abstract

We report on a boy with diabetes mellitus and a phenotype indicating *glucokinase* (*GCK*) insufficiency, but a normal *GCK* gene examination applying direct gene sequencing. The boy was referred for diabetes mellitus at 7.5 years old. His father, grandfather and great grandfather suffered type 2 DM. Several blood glucose profiles showed (BG) of 6.5–10 mmol/L L. After three years on neutral insulin Hagedorn (NPH) in a dose of 0.3 IU/kg/day haemoglobin A1c (HbA1c) was 6.8%. Treatment was changed to sulphonylurea 750 mg a day, and after 4 years HbA1c was 7%. At that time a multiplex ligation-dependent amplification gene dosage assay (MLPA) was done, revealing a whole *GCK* gene deletion. Medical treatment was ceased, and after one year HbA1c was 6.8%. This case underscores the importance of a MLPA examination if the phenotype of a patient is strongly indicative of *GCK* insufficiency and no mutation is identified using direct sequencing.

## 1. Introduction

Monogenic diabetes results from a mutation in a single gene. Most cases are autosomal dominant inherited, but may be recessive inherited, mitochondrial inherited, or be due to a *de novo *mutation [1]. Monogenic diabetes should be considered when diabetes is inherited through more than two generations. A common cause of monogenic diabetes, Maturity Onset Diabetes of the Young (MODY) is mutations in the glucokinase (*GCK*) gene (MODY2). MODY2 is characterized by moderately elevated fasting and postprandial blood glucose levels. Treatment is usually unnecessary [1]. Until recently, mutation screening has been restricted to denaturing high-performance liquid chromatography (dHPLC) or direct sequence analyses. However these methods do not detect large heterozygous deletion mutations. 

We report on a boy with diabetes mellitus and a phenotype indicating *GCK* insufficiency, but a normal *GCK* gene examination applying direct gene sequencing.

## 2. Case Report

**Figure 1:**
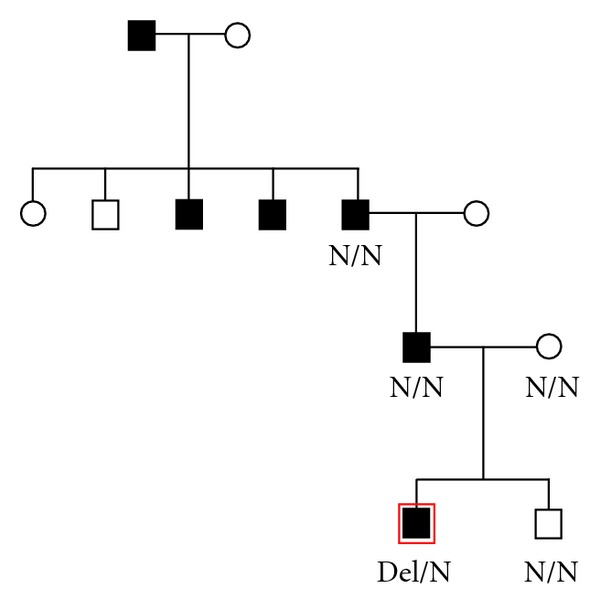
Pedigree of the family of the proband. The proband is marked with a red square. Filled symbols indicate family members with diabetes. The carrier of the *GCK *deletion mutation is marked with Del/N. Family members tested negative for the *GCK *deletion are marked with N/N.

**Figure 2:**
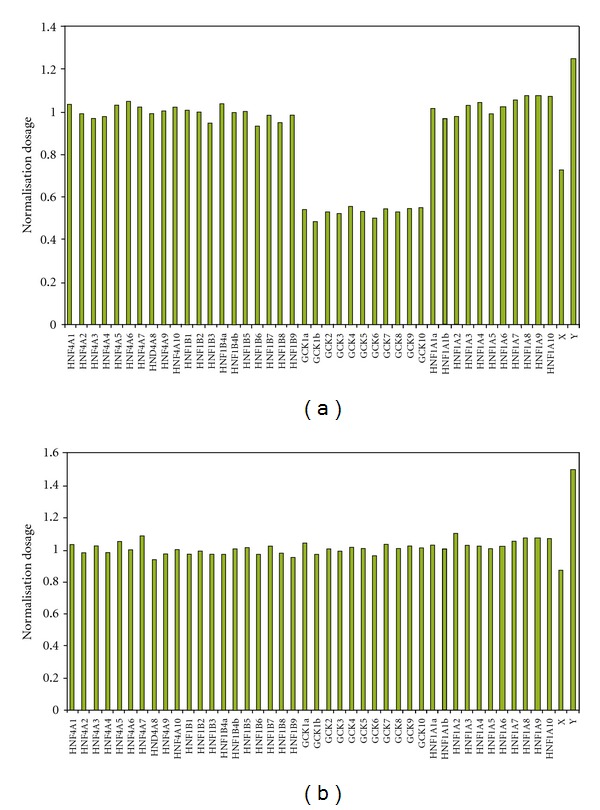
Detection of a whole *GCK* gene deletion (MODY2) by multiplex ligation-dependent amplification dosage assay (MLPA). Graphical representation of *HNF4A *(MODY1)*, HNF1A *(MODY3)*, HNF1B *(MODY5)*, GCK *(MODY2), Chromosome X, and Y probes normalised to controls in the proband (a) and one of the examined family members (b). All *GCK* probes are expressed in half dosage in the proband indicating a total *GCK *deletion.

The boy was referred for diabetes mellitus (DM) at 7.5 year old, with two fasting blood glucoses (BG) of 7-8 mmol/L and a HbA1c of 6.4%. He was born at term with a birth weight of 2800 g and a birth length of 51 cm. He was slightly retarded. An MRI of the brain was normal apart from an arachnoideal cyst at cisterna ambiens. The parents were nonconsanguineous. The father, grandfather, two of the grandfather's brothers, and the great grandfather were diagnosed with type 2 DM (Figure 1). The grandfather died at 54 years old. The pedigree indicated that the patient might have monogenic diabetes. However, gene sequencing of all *GCK* exons, intron-exon boundaries, and the promoter did not reveal any mutations. After several BG profiles of 6.5–10 mmol/L, the boy was treated with NPH insulin 8 IE in the morning (0.3 IE insulin per kg body weight). After three years on NHP 8 IE once a day, HbA1c was 6.8%. Stimulated C-peptide was 842 pmol/L, and the boy was tested negative for GAD65 and IA2 autoantibodies. The treatment was changed to sulphonylurea 750 mg a day. After four years on sulphonylurea, HbA1c was 7.0%. At that time a multiplex ligation-dependent amplification dosage assay (MLPA) revealed a whole *GCK* gene deletion (Figure 2). The *GCK* gene deletion was not identified in the parents and a brother, indicating that the deletion was a de novo mutation. The sulphonylurea treatment was ceased. One year later, HbA1c was unchanged 6.8%. An array comparative genomic hybridization of the whole genome did not reveal other mutations, and only the *GCK* gene was deleted. 

## 3. Discussion

Most *GCK* mutations are single nucleotide mutations, which can be detected by dHPLC or direct gene sequencing [2, 3]. *GCK* deletion mutations are very rare [4]. Until now a whole *GCK* deletion in nonsyndromic patients have only been reported one time [2]. In syndromic patients, whole *GCK *gene deletions have been reported in combination with multiple gene deletions [5, 6]. In our patient, the pedigree and the phenotype gave a strong suspicion of a *GCK* mutation, and a *de novo* mutation with a whole *GCK* gene deletion was detected by MLPA. The relative low birth weight can be explained by the mother not having the *GCK *mutation and the fetus having the *GCK *mutation, which indirectly decreases fetal insulin secretion and thereby fetal growth [7]. This case emphasizes the importance of gene dosage analysis by MLPA in patients suspected for a *GCK* mutation, and no mutation was identified applying direct sequencing.
